# Internal Mammary Artery Compression After Pectus Excavatum Repair Does Not Increase Risk of Hemorrhagic Complications in Pediatric Patients

**DOI:** 10.3389/fped.2020.619065

**Published:** 2021-01-05

**Authors:** Tamás Kovács, Gyula Pásztor, Anna Rieth

**Affiliations:** ^1^Unit of Pediatric Surgery, Department of Pediatrics, Albert Szent-Gyorgyi Clinical Center, University of Szeged, Szeged, Hungary; ^2^Department of Radiology, Albert Szent-Gyorgyi Clinical Center, University of Szeged, Szeged, Hungary

**Keywords:** complications, Doppler ultrasound, pectus excavatum, bar removal, internal mammary artery

## Abstract

**Objectives:** Minimal invasive repair of pectus excavatum (MIRPE) described by Nuss is the most popular correction nowadays of this deformity. During the introduction of the bars, they can hurt or compress the internal mammary arteries (IMA). The aim of this study was to observe the prevalence of IMA compression in children after MIRPE. Also, we examined if IMA obstruction increases the risk of complications at bar removal, and if these vascular changes are reversible.

**Materials and Methods:** All patients operated on pectus excavatum in our tertiary pediatric surgical center between 2013 and 2019 were involved in the study. Data of age, sex, number of bars and characteristics of the deformity were examined. IMA flow was checked by Doppler ultrasound (DUS) after MIRPE and after bar removal, too.

**Results:** Among 41 patients with mean age of 15.2 years there were 18 asymmetrical deformities, 23 sternal rotations. Mean pectus index was 4.01. After the Nuss procedure 7(9%) stenoses and 10(12%) occlusions of IMA were found on DUS. After bar removal 3 of 4 stenoses have resolved, but all examined occlusions (3/3) persisted. There were no complications during bar removals.

**Conclusion:** IMA compression after MIRPE in children is uncommon, and is not influenced by severity of deformity. Obstruction of these vessels does not increase the risk of hemorrhagic complications at bar removal. Data of larger cohort are needed to determine reversibility of these changes.

## Introduction

Pectus excavatum (PE) is the most common chest wall deformity, characterized by depression of the sternum and adjacent costal cartilages. It occurs in 1:400 live births, with a 1:4 male predominance. Since 1949 the surgical correction described by Ravitch has become widespread (1), until in 1998 Donald Nuss published a minimal invasive repair technique for PE (MIRPE) (2). Since then, this type of surgery has gained popularity, with some technical modifications during the years (3–9). Correction of position of the sternum is achieved by one or more shaped and retrosternally positioned bars. During the introduction of the Nuss bars, they can hurt or compress the internal mammary arteries (IMA). These vessels arise from the subclavian arteries, and descend in the thorax posteriorly and laterally to the sternum. During the Nuss procedure, the introducer and the bar pass through the intercostal space near this point, which may lead to injury, occlusion or erosion of these vessels.

Adult series have investigated the effect of the Nuss procedure on the IMA flow (10). Pre- and post-operative CT angiography was used to detect the patency of these vessels. In 44% uni- or bi-lateral obstruction was found. Our aim was to observe the changes in IMA flow in pediatric patients after minimal invasive correction of PE using Doppler ultrasound (DUS). We examined if obstruction of IMA increases the risk of complications at bar removal. Also, we have observed whether the changes in IMA flow are reversible after removing the Nuss bars.

## Materials and Methods

Data of patients operated between December 2013 and September 2019 in our pediatric surgical department were analyzed after Local Ethics Committee approval. All patients have had written informed consent. All patients operated in this period with PE were involved in the study. Routine preoperative evaluation included low-dose chest CT, echocardiography, and thoracic Doppler ultrasound (DUS) examination. Data were collected regarding age at time of surgery, sex, number of bars, preoperative Haller index, symmetry of deformity, sternal rotation, and IMA flow. Ultrasound was performed by pediatric radiologists in B-mode and in color Doppler using GE Logiq S8 (GE Healthcare, Chicago, IL, USA) Disturbances of IMA flow were described as stenosis or occlusion at site of the bar. Increased velocity of blood flow at this point was defined as arterial stenosis. Moreover, in color Doppler examination aliasing was noted. Occlusion was described when the blood flow was not detectable caudally from the bar. IMA flow was examined after implanting the bar and also after bar removal.

### Statistical Methods

Descriptive statistics are presented as mean and SD for continuous data and frequencies and percentages as categorical data. For the comparison of groups, Welch test (Student's unpaired *t*-test for unequal variances) was used for continuous variables and Fisher's exact test was used for categorical variables. Calculations were performed by SPSS for Windows software (version 26.0; SPSS Inc., Chicago, IL, USA). *P*-values < 0.05 were regarded as statistically significant.

## Results

Forty-one patients (36 male and 5 female) were involved in the study. Mean age was 15.2 ± 1.3 years at time of the operation (median age: 15.04 years, range 13–19 years). There were 23 (56.1%) symmetrical and 18 (43.9%) asymmetrical deformities, accompanied with 23 rotational deformities (5 left and 18 right rotational). Preoperative mean Haller index of the patients was 4.01 ± 0.98 (range 2.32–6.1). All patients had Nuss procedure with thoracoscopic guidance, two of them received two bars, while in 39 patients one bar was employed.

Eighty two IMAs (in 41 patients) were examined by pre- and post-operative thoracic DUS. Examination revealed patent vascular flow in 65 arteries (79%). Mean vascular flow of these unaffected arteries was 71.5 ± 38.42 cm/sec (range 31–140 cm/s) on the left, and 83.3 ± 31.06 cm/s (range 27–162 cm/s) on the right side.

Affected IMAs were noted as stenosis in seven arteries (9%), and occlusion in 10 arteries (12%). These obstructions were unilateral in 88%, and bilateral in 12%. Characteristics and data of patients are shown in Table 1.

**Table 1:** Characteristics and data of patients.

**Patients no**.	**Gender (M/F)**	**Age (years)**	**Haller index**	**Type of deformity (S/A)**	**Rotation (No/R/L)**	**Left IMA PSV (cm/sec)**	**Right IMA PSV (cm/sec)**	**Stenosis /Occlusion after MIRPE**	**IMA flow after bar removal**
1	M	15	5.2	S	No	224	87	Sten. l.s.	Sten. l.s.
2	F	13	5.3	A	R	60	82	Normal	Normal
3	M	16	4.8	A	R	131	123	Normal	Normal
4	M	16	4.5	S	No	113	61	Normal	Normal
5	M	15	2.8	A	No	54	27	Normal	Normal
6	M	16	4.3	A	L	90	103	Normal	Normal
7	M	16	4.2	A	R	117	120	Normal	Normal
8	M	14	3.3	A	R	0	58	Occl. l.s.	Occl. l.s.
9	M	14	5.9	A	R	77	89	Normal	Normal
10	M	15	3.0	S	No	63	69	Normal	Normal
11	M	17	4.9	S	No	85	111	Normal	Normal
12	M	16	6.1	A	R	69	139	Normal	Normal
13	M	15	4.1	A	R	75	81	Normal	Normal
14	M	16	3.0	A	L	50	72	Normal	Normal
15	M	17	3.9	S	R	140	162	Normal	Normal
16	M	16	3.7	S	No	62	50	Normal	Normal
17	M	14	3.6	S	No	37	60	Normal	Normal
18	M	14	2.8	S	R	52	177	Sten. l.d.	Normal
19	M	15	2.9	A	L	0	102	Occl. l.s.	Occl. l.s.
20	M	13	2.9	S	No	75	95	Normal	Normal
21	F	14	4.8	A	R	105	54	Normal	Normal
22	M	16	3.8	A	L	54	71	Normal	Normal
23	M	16	4.7	S	No	0	83	Sten. l.d., Occl. l.s.	Occl. l.s., Normal l.d.
24	F	16	4.3	S	R	100	113	Normal	Normal
25	M	14	3.7	S	R	48	160	Sten. l.d.	Normal
26	F	19	2.9	S	R	100	97	Normal	Normal
27	M	16	2.3	A	L	50	45	Normal	Normal
28	M	13	5.4	A	R	0	93	Occl. l.s.	–
29	M	15	4.0	S	No	35	58	Normal	–
30	M	14	3.7	A	R	31	0	Occl. l.d.	–
31	M	16	5.0	S	No	0	92	Occl. l.s.	–
32	M	16	3.3	A	R	177	60	Sten. l.s.	–
33	M	16	2.8	S	No	58	122	Normal	–
34	F	14	4.1	S	R	0	0	Occl. l.u.	–
35	M	14	4.1	S	No	62	205	Sten. l.d.	–
36	M	16	2.3	S	No	35	60	Normal	–
37	M	14	3.9	A	R	65	0	Occl. l.d.	–
38	M	14	5.0	S	No	55	32	Normal	–
39	M	16	4.0	S	No	0	113	Occl. l.s.	–
40	M	15	4.0	S	No	26	49	Sten. l.s.	–
41	M	17	4.0	S	No	67	82	Normal	–

We have compared the patients with affected and unaffected IMA flow, whether there is any difference among them in terms of age, sex, severity of deformity, symmetry, rotational deformity. As Table 2 shows there is statistically significant difference between the two groups only regarding age, but asymmetry, sternal rotation, higher Haller index do not elevate the risk of IMA compression.

**Table 2:** Comparison of patients with affected and unaffected IMA flow.

	**IMA flow unaffected**	**IMA flow affected**	***P***
Sex			0.636
M	22	14	
F	4	1	
Age			0.022
mean	15.54	14.67	
SD	1.334	0.976	
Haller-index			0.987
mean	4.012	4.007	
SD	1.0520	0.7896	
Symmetry			0.754
Yes	14	9	
No	12	6	
Rotation			0.754
No	12	6	
Yes	14	9	
Number of bars			0.128
1	26	13	
2	0	2	

So far the bars were removed from 27 of the 41 patients. Mean length between Nuss procedure and bar removal was 3.01 years (range 2.76–3.19 years). There were no complications observed during these operations. IMA flow was checked also after bar removal. Among the 27 patients there were six with IMA compression (four stenoses, three occlusions, in one patient bilateral compression). We have found that three stenoses have resolved, while one stenosis and all three occlusions were still noted on control DUS. In the other 21 patients, in whom the post- Nuss procedure DUS showed normal IMA flow also had normal flow on the US performed after bar removal.

## Discussion

Nuss procedure is the gold standard treatment for PE to correct the anatomical deformity of the chest wall. The operation is safe and gives perfect cosmetic result, however it is important to know that certain mild or serious complications can occur (3). The overall incidence of these complications is reported between 2 and 20% (11); however the incidence of severe complications is fortunately only 0.1% (8). Among the latter, injury of the heart (12), major vessels (12, 13), lung (14), diaphragm (15) can occur. Predisposing factors for cardiac injury are mediastinal adhesions due to previous cardiac or thoracic surgery, and the risk of this complication can be decreased with the use of thoracoscopy, sternal elevation and substernal dissection (3, 9, 16, 17).

Another source of hemorrhage can be the intraoperative injury of vessels, most frequently the IMA. As the IMA traverse posterolateral to the sternum, the introducer or the bar might injure it. This complication can also be minimized with the use of thoracoscope, when the bar or introducer can be followed under direct visual control (11, 17). As late on-set complication, hemothorax resulting from erosion of the IMA by the Nuss bar is published, too (18, 19). In these cases the bleeding was stopped by angiographic embolization of IMA (18) or ligation of the vessel *via* thoracotomy (19).

The introduced bar distending to the sternum can also compress the IMA-s, causing stenosis or complete obstruction in them. Yüksel et al. used pre and postoperative CT angiography (CTA) in adults to detect the change in IMA blood flow. In 44% of patients the blood flow was affected. Among these patients 33% bilateral, 46% unilateral obstruction, and 21% unilateral stenosis was observed (10).

In pediatric patients CTA is not advised to measure the blood flow in these vessels because of the high exposure to radiation. However, Doppler US can be perfectly used to investigate IMA flow in children. A tri-phasic curve with normal velocity shows normal flow, while elevated speed or non-tri-phasic curve means stenosis and lack of curve means complete obstruction.

According to our data IMA compression in pediatric patients after MIRPE is not common, in 79% of the cases the flow is unaffected. In 9% stenosis, while in 12% total occlusion of these vessels was detected. The alterations of the blood flow were unilateral in most cases (88%), and bilateral only in 12%. Interestingly, these changes were independent from the severity and characteristics of the deformity.

Serious, even fatal hemorrhagic complications are reported in literature at bar removal, too (11, 20). As the source of bleeding, the injury of heart, lung, aorta or sternal erosion are mentioned (11, 20–24) and it is related to mediastinal adhesions between these organs and the bar. Carlucci advises the use of thoracoscopy for bar removals in case of bar dislocation (22). Campos proposed to cover the serrated edges of the implants with a protective film at bar removal to minimize hemorrhagic complications (25). Toselli uses a safety string at all bar removals, and in case of bleeding a sponge or Sangstaken Blakemore catheter can be attached to it and can be dragged into the tunnel to control the hemorrhage (26). We have examined, whether removing those bars which were compressing the IMA-s increase the risk of intraoperative hemorrhage, and if these bar removals should be performed with extra precautions, for example, thoracoscopic control. Fortunately, so far there were no complications during these removals, and thus we don't advice any change in the operative technique in case of IMA compression.

As IMA is used in adult cardiac surgery for coronary artery bypassing grafting, it can be important to know if the changes in IMA flow are reversible after bar removal. In a pilot study among adults with bar removal after Nuss repair, Külcü published IMA occlusion in four of s patients (27). Therefore, he recommends preoperative evaluation of IMA patency in all patients with history of PE repair undergoing coronary bypass grafting. To prevent IMA compression by the Nuss bar, Sessar advices thoracoscopic mobilization of IMA at the level of bar implantation during MIRPE and pass the bar between the chest wall and the artery (28). On the other hand, this procedure might lead to adhesions and cause bleeding at bar removal (29). Cabrera recommends taking down the IMA-s from the intercostal space and placing pericardium membrane between the bar and the vessels, especially in patients with family history of coronary artery disease (30).

Our study is still in progress, but after removing more than half of the bars from our examined patients, it seems that while stenosis can be reversible (in 75% of stenoses the flow has normalized after ceasing of compression), occlusion is definitive, as flow was not detectable in any of these vessels after the bar removal. Naturally, data of a large multicenter cohort can give the exact answer, how much the circulation in these vessels can normalize after bar removal.

## Conclusion

Our data shows that IMA obstruction is not common in pediatric patients after MIRPE. Stenosis and occlusion is not influenced by severity of the deformity, sternal rotation or asymmetry. IMA compression associated with MIRPE does not elevate the risk of hemorrhagic complications at bar removal. Further studies on large cohort are needed to determine the reversibility of the changes in IMA flow after removing the pectus bars.

## Data Availability Statement

The original contributions presented in the study are included in the article/supplementary material, further inquiries can be directed to the corresponding author/s.

## Ethics Statement

The studies involving human participants were reviewed and approved by Scientific Ethic Committee of University of Szeged. Written informed consent to participate in this study was provided by the participants' legal guardian/next of kin.

## Author Contributions

TK and AR participated in collecting and interpreting of data. The manuscript was written by TK. The ultrasound examinations were performed by GP. All authors contributed to the article and approved the submitted version.

## Conflict of Interest

The authors declare that the research was conducted in the absence of any commercial or financial relationships that could be construed as a potential conflict of interest.

## Abbreviations

CTA: computerized tomography angiography
DUS: doppler ultrasound
IMA: internal mammary artery, internal thoracic artery
MIRPE: minimally invasive repair of pectus excavatum
PE: pectus excavatum
PSV: peak systolic velocity.
